# Application of normalisation process theory in understanding implementation processes in primary care settings in the UK: a systematic review

**DOI:** 10.1186/s12875-020-01107-y

**Published:** 2020-03-16

**Authors:** Lisa Huddlestone, Jessica Turner, Helen Eborall, Nicky Hudson, Melanie Davies, Graham Martin

**Affiliations:** 1 Department of Health Sciences, University of Leicester, George Davies Centre, University Road, Leicester, LE1 7RH UK; 2 School of Applied Social Sciences, De Montfort University, Hawthorn Building, The Gateway, Leicester, LE1 9BH UK; 3 Diabetes Research Centre, Affiliated with the Department of Health Sciences, University of Leicester, Leicester General Hospital, Gwendolen Road, Leicester, LE5 4PW UK; 4 The Healthcare Improvement Studies Institute (THIS Institute), University of Cambridge, Cambridge Biomedical Campus, Clifford Allbutt Building, Cambridge, CB2 0AH UK

**Keywords:** Primary care, General practice, Normalisation process theory, NPT, United Kingdom, Complex interventions

## Abstract

**Background:**

Normalisation Process Theory (NPT) provides a framework to understand how interventions are implemented, embedded, and integrated in healthcare settings. Previous reviews of published literature have examined the application of NPT across international healthcare and reports its benefits. However, given the distinctive clinical function, organisational arrangements and the increasing management of people with a wide variety of conditions in primary care settings in the United Kingdom, it is important to understand how and why authors utilise and reflect on NPT in such settings to inform and evaluate implementation processes.

**Methods:**

A systematic review of peer-reviewed literature using NPT in primary care settings in the United Kingdom (UK) was conducted. Eight electronic databases were searched using replicable methods to identify articles published between January 2012 and April 2018. Data were analysed using a framework approach.

**Results:**

Thirty-one articles met the inclusion criteria. Researchers utilised NPT to explore the implementation of interventions, targeting a wide range of health services and conditions, within primary care settings in the UK. NPT was mostly applied qualitatively; however, a small number of researchers have moved towards mixed and quantitative methods. Some variation was observed in the use of NPT constructs and sub-constructs, and whether and how researchers undertook modification to make them more relevant to the implementation process and multiple stakeholder perspectives.

**Conclusion:**

NPT provides a flexible framework for the development and evaluation of complex healthcare interventions in UK primary care settings. This review updates the literature on NPT use and indicates that its application is well suited to these environments, particularly in supporting patients with long-term conditions and co-morbidities. We recommend future research explores the receipt of interventions by multiple stakeholders and suggest that authors reflect on justifications for using NPT in their reporting.

## Background

Service innovations in healthcare settings are a deliberate attempt to introduce new interventions or practices, or to transform existing interventions or practices, for the purposes of assessing, improving, maintaining, promoting or modifying health and wellbeing [1]. These can include new techniques, such as a new way of treating an illness or condition, new technologies, such as a new medical device or IT system, or organisational changes, for example relating to behaviour or working practices [2–4]. Complex interventions are defined as *“interventions that contain several interacting components*” [5].

The theoretical and empirical literature demonstrates difficulties in implementing new complex interventions resulting from multiple crosscutting factors, including policy context, organisational context and change, professional identity and relationships, individual actions, and the dissemination and uptake of knowledge [6–10]. This evidence highlights that healthcare innovations are impacted by historical and present-day contexts, and the inter-relationships between and within individuals and organisational systems [11].

Several factors influence healthcare professionals’ efforts to engage with or implement interventions. These relate to the degree to which professionals view the intervention as valuable, the active division of interventional work and the allocation of roles, the fit of the intervention (and its implications for the organisation and distribution of work) with current practice or routines, and the degree to which organisational structures facilitate and support staff participation [12, 13]. Studies have also recognised the importance of measuring and demonstrating the impact of the new intervention, and accounting for issues such as resource constraints and staffing and service structures that may influence implementation [12, 14, 15]. This has led to the development of explanatory and predictive models that seek to aid analysis and understanding of the factors that influence the uptake, routinisation and sustainability of innovations. One such model is Normalisation Process Theory (NPT).

### Normalisation Process Theory

NPT is a middle-range sociological theory that conceptualises implementation, embedding, and integration of innovation in healthcare settings [16]. It provides ‘a set of sociological tools to understand and explain the social processes through which new or modified practices of thinking, enacting, and organising work are operationalised in healthcare and other institutional settings’ [16]. By emphasising the interactions between contexts (encompassing organisational and technical structures), actors (including individuals and groups), and objects (such as clinical practices and procedures), it facilitates examination and understanding of the translational gap between evidence, policy, and practice [17]. It focuses in particular on the work required of stakeholders to embed and normalise innovations in routine practice. More recently it has been used to account for the influence of the characteristics of innovations and contexts themselves in facilitating or impeding this work [18].

Initially developed as an applied theoretical model, the Normalisation Process Model (NPM) sought to assist the understanding and evaluation of factors that act as barriers or enablers for routine incorporation of complex healthcare innovations into practice. While the NPM explained factors that promote or inhibit the distribution of work among stakeholders and supportive resources, it did not address how stakeholders understand, engage with, and evaluate the innovation [19]. To overcome this, Finch et al. developed three further constructs to account for how stakeholders understand and make sense of practice, engage and participate with the innovation, and reflect on or appraise its effects [19]. Through this development, the NPM became NPT which was then further elaborated through an increasingly sophisticated account of the features of context (in particular its ‘elasticity’) and the intervention (its ‘plasticity’) that could themselves influence the viability of actors’ work to normalise newly introduced innovations [18]. Definitions of NPT’s key constructs and sub-constructs are summarised in Table 1.

**Table 1:** NPT constructs and sub-constructs as described by Finch et al. [19]

	Coherence	Cognitive Participation	Collective Action	Reflexive Monitoring
**Construct**	*The process and work of sense making and understanding that individuals and organisations undertake that promote or inhibit the routine embedding of a practice*	*The process and work that individuals undertake to promote engagement with the new practice*	*The work done by individuals and organisations to enact the new practice.*	*The work inherent to formal and informal appraisal of new practice, to enable assessment of advantages and disadvantages, developing users comprehension of the effects of a practice*
**Sub-constructs**	***Differentiation*** *Do stakeholders see this as a new way working?*	***Enrolment*** *Do the stakeholders believe they are the correct people to drive forward the implementation?*	***Interactional workability*** *Does the intervention make it easier or harder to complete tasks?*	***Systemisation*** *Will stakeholders be able to judge the effectiveness of the intervention?*
	***Individual specification*** *Do individuals understand what tasks the intervention requires of them?*	***Initiation*** *Are they willing and able to engage others in the implementation?*	***Skill set workability*** *Do those implementing the intervention have the correct skills and training for the job?*	***Individual appraisal*** *How will individuals judge the effectiveness of the intervention?*
	***Communal specification*** *Do all those involved agree about the purpose of the intervention?*	***Activation*** *Can stakeholders identify what tasks and activities are required to sustain the intervention?*	***Relational integration*** *Do those involved in the implementation have confidence in the new way of working?*	***Communal appraisal*** *How will stakeholders collectively judge the effectiveness of the intervention?*
	***Internalisation*** *Do all the stakeholders grasp the potential benefits and value of the intervention?*	***Legitimation*** *Do they believe it is appropriate for them to be involved in the intervention?*	***Contextual integration*** *Do local and national resources and policies support the implementation?*	***Reconfiguration*** *Will stakeholders be able to modify the intervention based on evaluation and experience?*

### Normalisation Process Theory’s use in implementing health service interventions

The use of NPT in health research is growing. Originally used to evaluate e-health and tele-health interventions, its use has spread to a diverse range of health-related settings and interventions [20, 21]. For example, a review by McEvoy et al. found that many authors endorse the use of NPT both in the analysis of wider implementation processes and in guiding recommendations for future implementation work [20]. In our research on the embedding of new practices around education for self-management for a long-term condition (Type-2 Diabetes), we were concerned with the work that individuals and groups have to undertake in order to embed innovation and for it to be sustained into routine practice; NPT pays attention to these dynamics with a contextual focus and robust theoretical basis. We therefore focussed on understanding how NPT has been operationalised in primary care in the United Kingdom (UK) to support the implementation and embedding of complex interventions.

### Rationale for this review

Researchers [20, 22] have advocated for an increased understanding of how NPT might be used to shape implementation processes in ways that promote integration and embedding of complex interventions. A recent qualitative systematic review by May et al. [21], has provided a valuable characterisation and exploration of the contribution of NPT to the implementation of healthcare interventions in variety of health systems. However, the complexity of developing and implementing healthcare interventions in systems with unique characteristics, such as the UK National Health Service (NHS), has not yet been an explicit focus.

Further attention has been drawn to the use of NPT in NHS primary care by our own development and evaluation of a package of practical advice and solutions to increase uptake of structured management education for Type-2 Diabetes Mellitus, by addressing barriers and enablers to uptake at patient, healthcare professional and organisational levels.

Primary care in the UK and the system within which it operates is distinctive from health systems in other developed countries, in terms of its clinical function, organisational arrangements, workforce complexion, and funding.

### Complexity of the UK primary health care system

Organisationally the NHS comprises many organisations and structures. Primary care providers deliver frontline contact for non-urgent physical and mental health complaints and conditions. These include general practitioners (GPs), pharmacists, dentists, and opticians. Where a condition requires specialist treatment or investigation, patients may be referred to another healthcare provider based in a hospital or the community.

While the NHS is traditionally thought of as a public entity, primary care organisations “straddle the public-private sector interface” [23]. For example, general practices are usually small businesses owned by GPs, which combine public sector funding (from general taxation), private ownership by GPs and personal profit. GP partners own practices and are subject to both the benefits and risks of investment and changing revenue streams; salaried GPs are employed by those practices (rather than by the NHS). In addition, a mixture of large commercial organisations or independent businesses provide pharmacy services. This organisational scene is volatile: larger businesses are increasingly encroaching on general practice, there are various arrangements for the provision of out-of-hours GP services, and primary care networks bring together individual practices into larger groupings with a view to increasing the flexibility and responsiveness of general practice, community pharmacy and other primary care services to patients [24].

Primary care in the UK faces major challenges, including increasing workloads, an ageing population, the need to manage increasing complex and multiple medical conditions in the community, and a focus on informing and providing choice to patients in healthcare decision-making [25]. These challenges are further compounded by reductions in capital investment, the need to increase access to care and communication between and within organisations, medical recruitment, and problems in the recruitment and retention of nursing staff [25].

The NHS performs well in managing chronic illnesses, such as diabetes, despite the fact that healthcare spending in the UK is lower than average for comparable countries [26]. This reduced spending has implications for the implementation of interventions, particularly embedding and adopting novel and often increasingly complex healthcare interventions, such as those set out by the *Long Term Plan* [24]. Adopters of an intervention play a significant role in the replication and translation of an intervention [27]. Achieving replication of an intervention may require staff to adapt and integrate the intervention into pre-existing or new ways of working, potentially requiring the development of new skills, or cultural change [27, 28]. Furthermore, contractual incentives for GP participation in budget holding, partnerships with other organisations to provide co-ordinated care and the sharing of financial risks have further changed the primary care system, meaning that the practicalities of intervention implementation require tailoring to and learning from the context [27, 29].

### Purpose of the review

Given this complexity, it is important to understand how NPT is being applied in order to contribute to an improved understanding of the complexities of intervention implementation, particularly in contexts and interactions that are resource constrained. Therefore, the purpose of this study was to investigate how NPT has been applied to primary care settings in the UK and to consider how it may be used to inform and assess implementation processes. To meet this aim, the objectives were to review the NPT literature in order to: a) understand what types of UK primary care interventions use NPT; b) explore how NPT was operationalised in practice in these examples; and c) examine how authors reflect on the use of NPT in UK primary care settings.

## Methods

A systematic review of the literature was conducted to explore the use of NPT in the development and evaluation of innovation in UK primary care settings, deploying systematic and transparent methods for literature identification, screening, and selection.

### Search and screening strategy

A systematic search of eight relevant databases (British Nursing Index, Ebsco Host Academic Premier, International Bibliography of Social Science, PubMed, Science Direct, Scopus, Web of Science, and Zetoc) was conducted for the period 1 January 2012 to 1 April 2018 for all relevant English-language publications. The search strategy is shown in Table 2.

**Table 2:** Search strategy and search terms

**Search terms**	
***1. Setting*** *“primary care” OR “family practice” OR “general practice”*	
***2. Study type*** “intervent*” OR “programme” OR “improv*” OR “evaluation”	
***3. Intervention activity*** *“Normalisation Process Theory” OR “NPT”*	
***4. Location*** “United Kingdom” OR “UK” OR “Great Britain” OR “Brit*” OR “Engl*” OR “Northern Ir*” OR “Scot*” OR “Wales” OR “Welsh”	
***Search Combinations*** *1 AND 2 AND 3 AND 4*	

Hand searching of the reference lists of included studies and relevant systematic reviews was conducted to identify further relevant papers. Citations were managed using RefWorks and divided equally between two authors (JT and LH). Titles and abstracts were screened against the defined inclusion criteria (Table 3). Where uncertainty arose as to whether a paper met the inclusion criteria, it was selected for full-text screening. All papers meeting the inclusion criteria were downloaded for full-text screening by the first and second authors to ensure consistency. Any differences were resolved by discussion among the authors.

**Table 3:** Inclusion criteria

**Inclusion criteria**	
• Method: Must use NPT • Setting: Primary care • Geography: United Kingdom • Time: 1 Jan 2012–1 Apr 2018 • Language: English • Document type: Empirical research • Availability: Full-text available	

### Data extraction

A structured data extraction instrument was developed. Authors (JT and LH) independently extracted data from all included papers using the tool and resolved any discrepancies in discussion, referring to the original papers. This enabled concerns or disagreements to be resolved through joint discussion. Extracted data comprised information on author(s), date of publication, study design and methods, sample and setting, topic, and implementation stage (shown in the populated form: see Table 4). Full text articles were then imported into NVivo 10 for analysis.

### Risk of bias assessment

The CASP checklist for qualitative research was used to assess 30 of the 31 articles. Among the three eligible articles describing randomised control trials (RCTs), two reported qualitative elements and were therefore assessed using the CASP checklist for qualitative research, and one was assessed using the CASP checklist for RCTs. Studies were categorised according to the checklist guidance.

### Data analysis

Analysis aimed to characterise and describe the use of NPT in UK primary care research, to explore how authors apply NPT throughout the implementation process, and understand how authors reflect on using NPT in UK primary care implementation research.

Initial analysis focussed on understanding and describing the characteristics (e.g. topic, methodology, methods) of the articles eligible for inclusion in this review. Next, we identified how NPT was applied and the stage of the implementation process it contributed to in each article. Frequencies were calculated for these and other study characteristics.

Articles were then reviewed to explore how NPT was operationalised in UK primary care research. A framework comprising four a priori items, relating to the four NPT constructs (*Coherence, Cognitive Participation, Collective Action* and *Reflexive Monitoring*) was chosen to sensitise researchers to the data. Analysis commenced with familiarisation with the data. This involved two authors (JT and LH) reading and re-reading the included articles. Data were individually extracted by JT and LH from any part of the paper detailing the use of any of the items.

Next, data relating to authors’ accounts of applying each NPT construct were extracted to examine whether these resonated with the description of the constructs put forward by the developers of NPT in their original exposition of the theory [16, 30]. Finally, all articles were reviewed to ascertain the presence of authors’ reflections on NPT use in primary care settings. Where these were identified, the researchers coded and organised these into broad themes.

## Results

### Search results

**Fig. 1:**
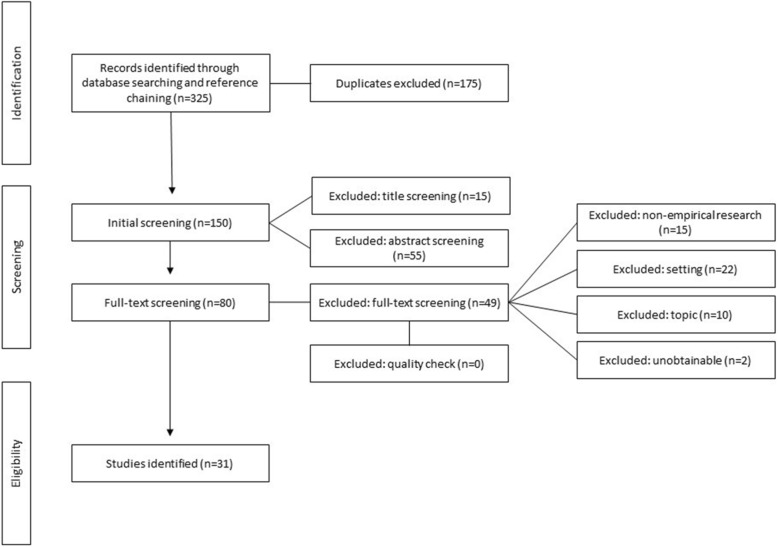
PRISMA diagram

The database and hand searches identified 325 articles, 31 of which were eligible for inclusion in this review. Fig. 1 details the process undertaken and provides information on the number and reasons for the exclusion of articles at each search stage.

### Risk of bias assessment

No papers were excluded during the quality assessment process: all 31 articles were deemed to be of sufficient quality according to our application of each checklist (see Additional file 1).

### Article characteristics

The diverse ways in which NPT has been used to develop and evaluate primary care interventions are summarised in Table 4. Briefly, twenty-nine studies were described in 31 articles, and published across 14 peer-reviewed journals.

**Table 4:** Characteristics of articles included in the review

Author and Date	Location	Design	Method	Setting	Sample	Topic	Implementation Stage
*Band* et al. [31] *(2017)*	*England*	*Mixed Methods*	*Semi-structured interviews; focus groups; synthesis of qualitative literature; quantitative systematic review*	*Primary care*	*Patients (n = 50);* *Patient interviewees (n = 16);* *HCP interviewees (n = 16)*	*Blood pressure*	*Development*
*Bayliss* et al. [32] *(2016)*	*England*	*Qualitative*	*Semi-structured interviews*	*Primary care*	*Patients (n = 11); GP (n = 8)*	*ME/CFS*	*Evaluation*
*Blickem* et al. [33] *(2014)*	*England*	*Qualitative*	*Ethnographic*	*Primary care*	*Patients (n = 20); Telephone Support Workers (n = 3)*	*Chronic kidney disease*	*Trial*
*Bouamrane and Mair* [34]. *(2013)*	*Scotland*	*Qualitative*	*Semi-structured interviews and focus groups*	*Primary care*	*GP (n = 25)*	*Electronic referral system*	*Evaluation*
*Bouamrane and Mair* [35]. *(2014)*	*Scotland*	*Qualitative*	*Semi-structured interviews and focus groups*	*Primary care*	*GP (n = 25)*	*Electronic referral system*	*Evaluation*
*Browne* et al. [36] *(2014)*	*Scotland*	*Qualitative*	*Semi-structured interviews and focus groups*	*Primary care Secondary care*	*Patients (n = 30); Carers (n = 20); Community Care Professionals (n = 39); Heart Failure Specialists (n = 22); Palliative Care Professionals (n = 4)*	*Chronic heart failure*	*Development*
*Buckingham* et al. [37] *(2015)*	*Scotland*	*Qualitative*	*Semi-structured interviews and group interviews*	*Primary care* *Community health*	*Patients (n = 32); Carers (n = 3); HCP (n = 28)*	*COPD*	*Trial*
*Carter* et al. [38] *(2016)*	*England*	*Qualitative*	*Semi-structured interviews and focus groups*	*Primary care*	*GP (n = 13); PN (n = 9); Practice Managers (n = 5); Practice Administrators (n = 23)*	*Patient feedback*	*Evaluation*
*Coupe* et al. [39] *(2014)*	*England*	*Qualitative*	*Semi-structured interviews*	*Primary care*	*Case managers (n = 6); Supervisors (n = 5); GP (n = 15)*	*Collaborative care: Depression*	*Evaluation*
*De Brún* et al. [59] *(2015)*	*Europe (Austria, England, Greece, Ireland, Netherlands, Scotland)*	*Documentary review*	*Mapping*	*Primary care*		*Cross-cultural communication*	*Development*
*Farr* et al. [40] *(2018)*	*England*	*Mixed-methods*	*Semi-structured interviews; patient survey; e-consultation data*	*Primary care*	*Primary care practice stakeholders [GP (n = 10); Nurse Practitioner (n = 1); Practice Managers(n = 6); Practice Administrators (n = 6)]; Patients (n = 75)*	*e-consultation*	*Evaluation*
*Grant* et al. [41] *(2017)*	*England*	*Qualitative*	*In-depth interview*	*Primary care*	*GP (n = 17); Practice Managers/Administrators (n = 9); Practice Pharmacists (n = 3)*	*Prescribing*	*Evaluation*
*Hoskins* et al. [42] *(2016)*	*Scotland*	*Mixed-methods*	*Patient data, semi-structured interviews (practice nurses and patients)*	*Primary care*	*PN (n = 10); patients (n = 14)*	*Asthma management*	*Trial*
*Kennedy* et al. [43] *(2014)*	*England*	*Mixed-methods*	*Semi-structured interviews and survey data*	*Primary care*	*Patients (n = 24) and Stakeholders from 31 practices including GP; Nurses,* *Practice manager and Administrators*	*Long-term conditions*	*Evaluation*
*Kennedy* et al. [44] *(2014)*	*England*	*Qualitative*	*Semi-structured interviews*	*Primary care*	*PN (n = 11); Assistant Practitioners (n = 1)*	*Long-term conditions*	*Evaluation*
*Knowles* et al. [45] *(2013)*	*England*	*Qualitative*	*Semi-structured interviews*	*Primary care*	*Psychological Well-being Practitioners (n = 6); PN (n = 17)*	*Collaborative care: Mental health co-morbidity*	*Evaluation*
*Ling* et al. [46]* (2012)*	*England*	*Qualitative*	*Case-study; semi-structured interviews; observations; semi-structured questionnaire, and documentary analysis*	*Primary care Secondary care* *Community health*	*Staff stakeholders (n = 213)*	*Integrated care*	*Evaluation*
*Lionis* et al. [59] *(2016)*	*Europe (Austria, England, Greece, Republic of Ireland, Netherlands)*	*Qualitative*	*Stakeholder (JT PLA Style Focus Group)*	*Primary care*	*Total: n = 304 governmental and non-governmental agencies* *England n = 9.* *Total: Stakeholders (n = 78): England: Migrant Community stakeholders (n = 7); GP (n = 1), Interpreter (n = 1), Policy Maker (n = 1)*	*Guidelines and training initiatives (G/TIs) are available to support communication in cross-cultural consultations*	*Development*
*Martindale* et al. [47] *(2017)*	*England*	*Qualitative*	*Semi-structured interviews*	*Primary care*	*GP (n = 7); PN (n = 5); Community Pharmacist (n = 5); Practice Pharmacist (n = 4); Practice Administrator (n = 2); HCA (n = 1); Patients (n = 5)*	*Prevention of acute kidney injury*	*Evaluation*
*Morden* et al. [48] *(2015)*	*England*	*Qualitative*	*Ethnographic*	*Primary care*	*GP (n = 9);* *PN (n = 4)*	*Osteoarthritis*	*Evaluation*
*Morris* et al. [49] *(2016)*	*England*	*Qualitative*	*Semi-structured interviews*	*Primary care*	*GP (n = 12); PN (n = 8); Pharmacists (n = 12); Patients (n = 10)*	*Acute kidney injury*	*Evaluation*
*O’Donnell and Kaner* [50]. *(2017)*	*England*	*Qualitative*	*In-depth, semi-structured interviews*	*Primary care*	*GP (n = 14)*	*Brief alcohol interventions*	*Evaluation*
*Ong* et al. [51] *(2014)*	*England*	*Qualitative*	*Ethnographic*	*Primary care*	*GP (n = 10); PN (n = 5)*	*Osteoarthritis*	*Evaluation*
*Porter* et al. [52] *(2016)*	*Wales*	*Qualitative*	*Semi-structured interviews and focus groups*	*Primary care*	*GP (n = 31); PN (n = 2); Practice Manager (n = 10)*	*Emergency admission prediction*	*Evaluation*
*Reeve* et al. [53] *(2016)*	*England*	*Qualitative*	*Ethnographic*	*Primary care*	*HCP; Commissioners, and Patients. (n = unspecified)*	*Mental health*	*Evaluation*
*Reeve* et al. [54] *(2018)*	*England*	*Quantitative*	*Survey*	*Primary care*	*Nurse Prescriber (n = 234); GP (n = 97); Pharmacist (n = 88)*	*Polypharmacy prescribing*	*Development*
*Ricketts* et al. [58] *(2016)*	*England*	*Qualitative*	*Semi-structured interviews*	*Primary care*	*GP (n = 9); PN (n = 13); Reception Staff (n = 7).*	*Sexual health*	*Evaluation*
*Rostami* et al. [55] *(2018)*	*England*	*Qualitative*	*Semi-structured interviews*	*Primary care Secondary care*	*Primary Care Pharmacists (n = 2); Hospital Pharmacists (n = 9); Hospital Nurse (n = 3); Clinical Auditor (n = 1)*	*Prescribing safety*	*Evaluation*
*Stevenson* [56]. *(2015)*	*England*	*Qualitative*	*Semi-structured interviews and focus groups*	*Primary care*	*Patients (n = 50); Practice Staff (n = 7); Stakeholders (n = 11)*	*CPRD implementation*	*Evaluation*
*Teunissen* et al. [61] *(2017)*	*Europe (Austria, England, Greece, Republic of Ireland, Netherlands)*	*Qualitative*	*Ethnography*	*Primary care*	*66 Stakeholders [GP (n = 14); PN (n = 8); Policy Makers (n = 12); Administrators (n = 6); Trainers (n = 4); Interpreters (n = 4); Migrants/Migrant Representatives (n = 18)]*	*Cross-cultural communication*	*Evaluation*
*Webster* et al. [57] *(2016)*	*England*	*Qualitative*	*Semi-structured interview*	*Primary care*	*Patients (n = 4); GP (n = 5); PN (n = 3); HCA (n = 1); mental health gateway worker (n = 1)*	*Collaborative care*	*Evaluation*

*GP* General Practitioner

*HCA* Health Care Assistant

*HCP* Health Care Professional

*PN* Practice Nurse

### Date of publication and geography

**Fig. 2:**
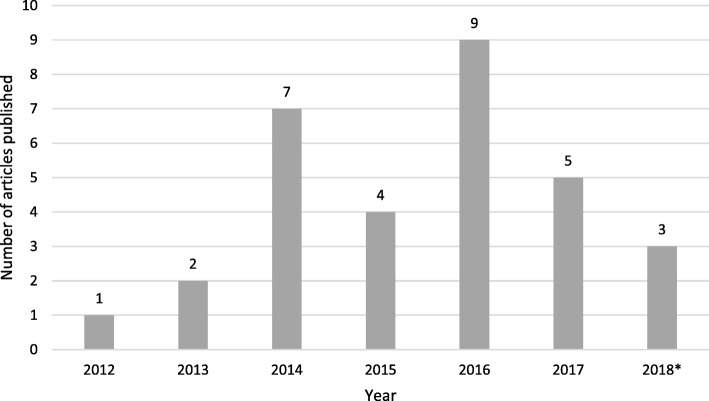
Number of articles reporting NPT use in UK primary care settings, by year. *to 1 April 2018

Most papers were published towards the end of the search period: from 2016 onwards (Fig. 2). Twenty-eight articles reported studies conducted within the UK only (England *n* = 22, Scotland *n* = 5, and Wales *n* = 1) [31–58]. The three remaining papers, which concerned cross-cultural communication, were conducted in European countries in addition to the UK (England and Scotland) [59–61].

### Study design and methods

Qualitative research designs were most frequently reported (*n* = 25), followed by mixed-method studies (*n* = 4). Quantitative and document analysis designs were utilised once each. Among the 25 articles using qualitative methods only, ten used semi-structured interviews [32, 39, 44, 45, 47, 49, 50, 55, 57, 58]; one utilised focus groups [60]; seven involved a combination of semi-structured interviews and focus groups [34–38, 52, 56]; six were ethnographic studies [33, 46, 48, 51, 53, 61]; and one used in-depth interviews [41]. The mixed-methods studies used varying combinations of semi-structured interviews, focus groups, survey data, patient data, and narrative and systematic reviews of the literature [31, 40, 42, 43]. The document analysis article adopted a mapping approach and the quantitative paper utilised a cross-sectional survey [54, 59].

### Study setting and sample

While all 31 studies were located within primary care settings, four studies also included secondary care, community health services, or a combination of these [36, 37, 46, 55]. Samples in the studies comprised patients, carers, healthcare professionals, administrators, commissioners, policymakers, and government and non-government agencies. Sample sizes ranged from 12 to 419 participants.

### Operationalising normalisation process theory in UK primary care

#### Implementation stage

The majority (*n* = 23) of studies reported using NPT in the pilot evaluation of interventions only [32, 36–41, 43–53, 55–58, 61], while five chose NPT as a framework for developing interventions [31, 36, 54, 59, 60], and three used the theory in RCTs [33, 37, 42].

#### Primary care topics under investigation

Thirteen articles reported the use of NPT to investigate interventions targeting long-term conditions [31–33, 36, 37, 42–45, 48, 51, 53, 57]. Conditions included hypertension, chronic fatigue syndrome / myalgic encephalomyelitis (CFS/ME), heart failure, kidney disease, chronic obstructive pulmonary disease, asthma, long-term conditions in general, and mental health conditions. Five articles investigated health service utilisation and referral using NPT [34, 35, 46, 52, 56]; six focussed on patient communication and consultation [38–40, 59–61]; two articles used NPT to investigate health promotion interventions [50, 58]; and five articles concerned medication management and prescribing [41, 47, 49, 54, 55]. Most studies (*n* = 26) applied NPT to evaluate interventions at various stages of the evaluation process [32–35, 37–53, 55–58, 61]. However, five articles reported on the use of NPT in developmental studies. These proposed interventions targeted the treatment of chronic heart failure, policy guidelines and training initiatives that support communication in cross-cultural primary care settings, prevention of acute kidney injury in patients with complex care needs, and the self-management of hypertension [31, 36, 49, 59, 60].

### Author rationale for NPT use

#### To aid design of instruments and procedures

The remaining eight studies used NPT prospectively, to inform the design of data collection tools and procedures [36, 37, 40, 48–51, 54]. Among these, NPT was utilised in the design of interview guides (*n* = 6) [36, 37, 40, 49–51], in the conception of interview and observational schedules (*n* = 1) [48], and in the development of a quantitative survey (*n* = 1) [54]. Seven of these studies also used NPT in the analysis phase [36, 37, 40, 48–50, 54].

#### To aid analysis

NPT was utilised to fulfil a number of functions. Twenty-three studies used NPT only in the analysis phase to frame and organise their findings [31–35, 38, 39, 41–47, 52, 53, 55–61]. Among these, three studies combined NPT with other theoretical frameworks to design, evaluate, and further refine interventions. One combined NPT with service co-production theory in order to understand how health professionals and patients co-implement, use, and experience an e-consultation system [40]. Another used NPT to further analyse intervention components identified by the COM-B model in the identification of potential implementation issues and development of a self-management intervention for hypertension [31]. The third combined NPT with participatory learning and action research techniques to generate stakeholder perspectives [60].

#### To aid implementation planning

Studies were reported to have used NPT to identify and explore issues related to intervention implementation, embedding, and sustainability. Thirteen studies used NPT to explore implementation issues [32, 39, 45, 49, 56–58, 61]: for example NPT assisted Bayliss et al. in understanding the processes and work required to implement and sustain the use of a CFS/ME training resource [32]. Eleven studies evaluated the impact of an innovation in terms of delivery and engagement [31, 33, 37, 38, 40–43, 47, 50, 51, 53, 55, 59]. For instance, Bouamrane et al. used NPT to interpret factors identified as facilitating and challenging the work of GPs during patient consultations, and to understand stakeholder perspectives on the implementation of a primary care e-referral system [34, 35]. Seven studies used NPT prospectively to explore the behavioural change required ahead of implementation [34–36, 44, 46, 48, 52, 54, 60]. One such example is Browne et al., who utilised NPT to focus attention on patients and carers’ work of managing a terminal condition and to characterise healthcare professionals’ responses to patients’ palliative care needs [36].

### Selection and application of NPT constructs

#### Frequency of construct occurrence

**Fig. 3:**
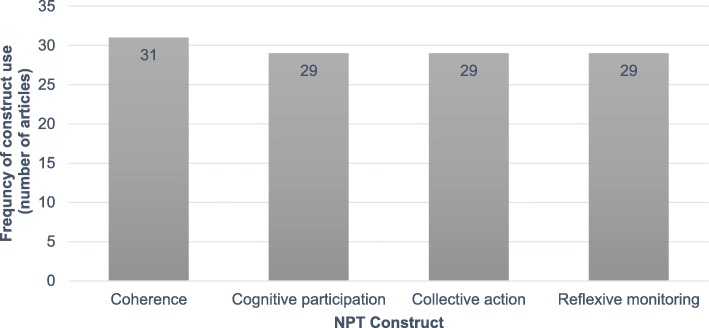
Frequency of NPT construct use by included articles

Figure 3 shows the number of times each of the four NPT constructs was used across the 31 included articles. All four NPT constructs (*Coherence*, *Cognitive Participation*, *Collective Action*, and *Reflexive Monitoring*) were operationalised in 29 articles [31–40, 42–51, 53–61]. The remaining two articles applied the construct of *Coherence* only [41, 52].

#### Alignment with NPT constructs and definitions

All articles aligned with the understanding of the four NPT constructs (C*oherence*, *Cognitive Participation*, *Collective Action*, *Reflexive Monitoring*) put forward by Finch et al. and May and Finch [16, 19]. However, some variation was evident in the extent to which studies defined the constructs used, and in whether and how the constructs were tailored to the intervention under investigation. For example some studies, such as Browne et al., modified the constructs to render them more relevant to the work of the intervention [36]. Modification in this sense involved the detailed tailoring of the NPT constructs to the work involved advanced cardiac failure care. Morden et al., meanwhile, noted that stringent application of the NPT constructs may influence the focus of the data collected, its analysis, and ultimately the findings [48]. To overcome this tension, the authors used NPT to sensitise them to the NPT constructs, but went beyond them in their analysis to allow the exploration of themes and topics outside of the scope of NPT [48].

The majority of articles reported the construct of *Coherence* as the extent to which study participants made sense of, and had a clear knowledge and understanding of the intervention [33, 34, 36–39, 43, 51, 52, 56]. However, other authors chose to modify the definition of *Coherence*, to include how interventional knowledge differed from and fitted with current practice [32, 51, 52, 59].

Articles identified *Cognitive Participation* as the extent to which participants bought into the intervention [33, 34], engaged with it [33, 37, 38, 44, 53, 56, 58], and committed to it [32, 37, 56]. *Collective Action* related to the allocation of organisational and personal resources to interventions [32, 34], how the intervention was operationalised [32–34, 37, 39, 58], and the definition of roles and responsibilities [34]. Authors constructed *Reflexive Monitoring* as the extent to which the interventions were subjected to appraisal and evaluation [32, 33, 37–39, 44, 58], assessments of interventional impact [34, 58], and processes of reflection, learning, and refinement to ensure sustained change [37, 38, 44, 58].

#### Application of NPT sub-constructs

Three articles [31, 41, 43] utilised all 16 sub-constructs of NPT. Band et al. applied all of the constructs and sub-constructs for the analysis of behaviours at the individual and organisational levels to identify appropriate behavioural change techniques in the development of an intervention addressing the management of hypertension [31]. Similarly, Kennedy et al. [43] used all the sub-constructs to evaluate the implementation of a self-management support approach intervention across patient, professional, and organisational groups, and Grant et al. [41] identified the impact of the intervention on the different participating general practices and their adoption of the innovation.

NPT was used by authors to fulfil a variety of functions. The majority of studies used the constructs of NPT to support analysis (*n* = 19) [31–35, 37, 41–45, 47, 53, 55–59, 61]. In addition, studies also used NPT in data collection (*n* = 12) [36–40, 48–52, 54, 60], and finally, NPT was utilised as tool to sensitise researchers to concepts relevant to the interpretation of data (*n* = 1) [46].

### Use and adaptation of NPT

NPT allowed for the identification of multiple influences across individuals and organisations, which both challenge and enable intervention implementation, embedding, and sustainability. A strength of NPT is that it takes account of the inter-relationship between social and structural constraints and individual agency – something which may be missed when individual behavioural change theories are used [50]. However, one paper suggests that focussing on the individual and collective agency of actors who are *delivering* the intervention risk neglecting the agency of those who *receive* the intervention – particularly at a patient or service user level [44]. The use of additional theories may also extend aspects of NPT to enable a better understanding how interventions are received, operationalised, and regarded by all stakeholders within a service. For example, Band et al. used NPT to characterise the everyday use of a blood pressure self-management intervention [31]. Combining behavioural analysis (NPT and the Behaviour Change Wheel), intervention planning (including the evidence base), and logic modelling, the authors claimed they achieved an in-depth and multifactorial understanding of the barriers and facilitators most relevant to the intervention and the user population [31].

### Authors’ reflections on NPT use

The authors of twenty-five articles reflected on their use NPT [31–45, 47–53, 55–61]. The majority of these articles (*n* = 16) reported only advantages of using NPT, while three reported only disadvantages, and six reflected on both advantaged and disadvantages. Following coding, authors’ reflections were organised into 12 themes, shown in Table 5 with example quotations.

**Table 5:** Author-reported reflections on using NPT in primary care setting

Author reflection	Example quotation
Identification of where the intervention addressed potential implementation issues [31, 37, 41, 49, 50, 53].	*“NPT helped to illuminate the context and localised systems approach that may need to be adopted to work with local stakeholders to implement sick day guidance”* [49].
Identification of acceptability, variations in implementation, and barriers to and feasibility of completing the intervention work in specific contexts [31, 34, 36, 38, 44, 47, 48, 55–57, 59, 60].	*“It is essential to understand the dynamic process of adaptation as an integral part of implementation and routinization, and to assess its contribution to eventual longer term outcomes (positive and negative)”* [48].
Useful way of understanding the experience of the implementation of innovation, from multiple perspectives [33, 38, 42, 45, 51, 57, 59, 60].	Perception that NPT facilitated appreciation of *“beliefs and opinions of people with different sociocultural status and educational background”* [60].
Provides a uniform interpretation scheme for the different views and beliefs of a diverse group of stakeholders [60].	
Refinement of intervention ahead of full trial [37, 53].	*“We have highlighted the use of Normalisation Process Theory to support development, and not just implementation, of a complex intervention”* [48].
Ability to complement other theories and frameworks [31, 40, 43].	*In adopting this approach, the intervention was grounded “in an in-depth understanding of the barriers and facilitators most relevant to this specific intervention and user population”* [31].
Disagreement over the operationalisation of NPT constructs [33, 38, 52, 58].	“*Whilst NPT is presented as a temporal process, analysis showed that many participants experience the constructs of NPT simultaneously*” [28].
Requires prior awareness of stakeholders and context in order to sensitise to the constructs [59].	“*We acknowledge that research teams found it difficult to answer some of the 16 sensitizing questions without knowing which stakeholders or sites were going to be involved with the implementation work*” [53].
Lacks consideration of the patient perspective on and/or role in implementation [40, 44].	“*There is a need for greater consideration in implementation theory of the importance of the patient role and the implementation work they need to do*” [43].
Places insufficient emphasis on those who receive complex interventions [43].	
Risk of artificially imposing (“shoehorning”) constructs onto data collection and analysis [48].	“*One tension in utilising such an approach is that it can influence the focus of the data collected, subsequent analysis, and the findings. But as detailed in the methods section we took steps to ensure themes, issues and topics which sat outside of the scope of NPT could be explored and accounted for*” [48].
Potential for cross-over of NPT constructs, differentiation of the four elements of the NPT framework [27, 32, 46, 55].	*“…understanding of the obstacles and drivers associated with embedding real-time feedback in general practices has been enhanced by organising qualitative data according to NPT constructs. … it is important to note that all four NPT constructs operated and were experienced concurrently*” [38].

#### NPT as a facilitator of understanding

In general, authors considered NPT advantageous in its recognition of the importance of context, identification of implementation enablers, as well as challenges and variations in the adoption and implementation of innovations [31, 34, 36, 38, 41, 44, 47, 49, 50, 55–57, 59, 60], particularly in the evaluation stage. For example, Bayliss et al. reported that NPT revealed that owing to a large number of barriers the CFS/ME intervention was not feasible, and concluded that *“time pressures and competing priorities meant that some GPs failed to engage with the training module (cognitive participation). When the module was completed, many GPs stated that it was not feasible to retain even the key messages as they saw so few patients with the condition”* [32]*.*

Strengths were also identified in relation to the use of NPT in the feasibility testing and refinement of innovation, in advance of full trial [31, 34, 36–38, 44, 47, 53, 55–57, 59, 60]. The use of NPT was also considered to facilitate researcher understandings of multiple stakeholder perspectives on innovation implementation [33, 38, 42, 45, 51, 57, 59, 60], using a consistent unifying framework [60]. For example Lionis and colleagues perceived that NPT facilitated appreciation of *“beliefs and opinions of people with different sociocultural status and educational background”* [60].

#### Bringing together NPT and other theories and frameworks

Three articles considered a major strength of NPT to be its ability to be used in conjunction with other theoretical frameworks [31, 40, 43]. Kennedy et al. considered that using the Consolidated Framework for Implementation Research in conjunction with NPT assisted in interpreting the wider context—and the tensions between policy and practice [43]. Farr and colleagues utilised service co-production processes theory in addition to NPT to analyse staff and patients’ initial expectations, interactions with and experiences of the intervention, and their subsequent perceptions resulting in satisfaction/dissatisfaction [40].

Finally Band et al. used NPT and two other approaches to theoretically model an intervention [31]. The authors mapped behaviour change techniques in the intervention onto the Behaviour Change Wheel and NPT frameworks, and developed a logic model. Using NPT, the authors were able to identify where the intervention addressed potential issues in implementation, link patient and health professional behaviour, and ascertain how the behaviours and their determinants mapped onto both psychological and sociological theoretical frameworks. In adopting this approach, the intervention was grounded “*in an in-depth understanding of the barriers and facilitators most relevant to this specific intervention and user population*” [31].

#### Challenges and complexity

Alongside these strengths, however, authors encountered several challenges. Firstly, authors reported the cross-over of NPT constructs, differentiation of the four elements of the NPT framework [33, 38, 52, 58] and the potential for ‘*shoehorning*’ data into the constructs [48] as problematic. Some suggested that these challenges were related to the non-linear nature of implementation. Indeed, Blickem et al. wrote that, “*Whilst NPT is presented as a temporal process, analysis showed that many participants experience the constructs of NPT simultaneously*” [33]. Second, some authors considered NPT to lack emphasis on those receiving the intervention, in particular at patient level [40, 43]. For example, Farr et al. suggested that “*service co-production theory and touchpoints can extend NPT through focussing on how technologies change the service process and interactions between patients and staff*” [40]. Kennedy and colleagues found “*that an NPT framework does not place ‘sufficient emphasis on those who receive complex interventions, especially when the ‘service user’ is referred to as a ‘partner in care*’“ [43]. Finally, de-Brún and colleagues reported challenges in prospectively using NPT: “*We acknowledge that research teams found it difficult to answer some of the 16 sensitizing questions without knowing which stakeholders or sites were going to be involved with the implementation work*” [59].

## Discussion

### Key findings of this review

The key findings of this review are that: (i) NPT provides an effective and flexible method for understanding a diverse variety of interventions implemented in UK primary care settings; (ii) NPT offers researchers the tools to understand the theoretical and practical challenges of implementation design and evaluation across, and within, complex health systems, such as UK primary care; (iii) NPT provides a constructive framework for explaining critical implementation processes for interventions focussed on the management of chronic health and multi-morbid conditions; and (iv) NPT appears helpful in understanding the implementation and evaluation of interventions in resource-constrained contexts.

This review identified 31 articles reporting on 29 studies of complex healthcare interventions and implementation processes in UK primary care settings. Eighteen [4, 32–35, 37, 39, 41, 43, 44, 46–48, 50, 51, 56–58, 61] of the articles identified in this review were included in a systematic review of 130 papers detailing NPT use in feasibility studies and process evaluations of complex health care interventions, authored by May et al. [21]. The articles in this review used NPT to evaluate, and to a lesser extent develop interventions. All but one of the articles used qualitative methods to collect and analyse data in ways that facilitated intervention and implementation planning and the identification of the dynamics of embedding interventions at the individual and organisational level.

As found in previous reviews [20, 21], the use of NPT in primary healthcare settings has largely focussed on feasibility and process evaluation studies. Among the articles in this review exploring NPT use in UK primary care settings, none used NPT to explore the entire implementation course of an intervention. However, this is not uncommon; May et al. [21] in their recent review identified only one longitudinal study [62].

Our findings indicate that NPT provides a consistent representation and explanation of the processes of intervention implementation in UK primary care, irrespective of the focus of the intervention. This suggests that authors are able to use NPT flexibly to depict theoretical and practical problems in intervention design and evaluation. Furthermore, the use of NPT, in either combination or isolation, is seen by authors to offer valuable contributions to the implementation of interventions targeting long-term conditions or multiple morbidity within a complex and resource-constrained system.

Despite a number of authors experiencing challenges with operationalising constructs, this review indicates that NPT constructs can be harmonised to inform prospective evaluation studies and this is demonstrated by the integration of NPT with other theoretical models [31, 40, 43]. Furthermore, this review has identified that NPT is able to move beyond organisational interventions, to those that seek to mobilise a range of stakeholders in various primary care settings, through the successful explanation of the outcomes of such intervention studies.

The flexibility of NPT is further highlighted by the use of constructs as sensitising concepts about implementation, with findings related back to the underpinning theory [41] and by the shaping of analytical frameworks to drive implementation refinement [43, 44]. In McEvoy et al.’s review, the authors cautioned of the danger of applying NPT as a “*conceptual straitjacket*” [20]. In this review, we have identified articles offering similar cautions. Nevertheless, the fact that findings may fall outside NPT’s constructs should not be considered problematic in itself, since the originators of NPT suggest that the theory can be deployed in multiple ways [17], and should be used heuristically rather than mechanistically. The use of NPT in implementation studies provides researchers with a holistic understanding of routines of new practice, as well as the implied and inferred activities that occur in making sense and apportioning meaning of interventions.

In contrast to the review by McEvoy and colleagues [20], this paper highlights the use of NPT prospectively, both in terms of intervention development and in the design of data collection and analytical instruments. In common with findings in the recent systematic review by May et al. [21], several authors considered NPT to lack consideration of the patient perspective or the perspective of those receiving, rather than delivering, the intervention. The originators of NPT do not restrict its focus to professional activity; indeed, Carl May and colleagues have explicitly applied it to the burden placed by the treatment of disease on patients and carers in developing the concept of ‘minimally disruptive medicine’ [63, 64]. The exclusion of patients and carers from NPT-informed analysis may arise because they are not centrally involved as stakeholders in implementation processes. It also, however, appears to reflect a view among some researchers that the theory pertains primarily to *professional* work. Finally, this review points to an increased awareness of the use NPT in whole-system analysis, with a number of articles reporting the inclusion of multiple stakeholder perspectives.

### Limitations

While a comprehensive search was undertaken, it is possible that some studies were missed, particularly if they did not explicitly state that they took place in primary care. However, it is debatable whether this would have influenced the overall findings. It is also important to note that our search strategy included only the English spelling of ‘Normalisation’ (as in the original usage) and as such, those articles choosing to use the American English spelling (Normalization) will not have been included in those articles retrieved. However, it is likely that at least some of these missed published articles, using the American spelling of ‘normalisation’, will have been retrieved using the abbreviation ‘NPT’ in our search. The a priori analysis plan in this study included three distinct phases. Firstly, we identified and characterised studies that had utilised NPT in the development and evaluation of interventions in primary care. Next, the fidelity of NPT constructs and sub-constructs use by authors was explored. Finally, we focussed on authors reflections on NPT use in UK primary care settings. While other researchers may be able to replicate this approach to reviewing the NPT literature, it is to some extent an inherently subjective process, and differing experiences, backgrounds and interests may mean that alternative conclusions may be drawn. While our findings may add to the evidence base concerning NPT and its use in healthcare, this review covers only UK-based primary care studies, and so extrapolation to other healthcare systems and settings may be limited.

The majority of included articles lacked description of the researcher-participant relationship, including bias and reflexivity, and while authors generally stated that ethical approval had been obtained, discussion of ethical or study-related considerations were limited. Limited reporting of these issues may be related to restrictions in publication word counts. Researchers might also wish consider ways to include reflexivity in their reporting of implementation studies. Authors’ rationales for the use of NPT was also lacking, meaning that it was not possible to ascertain if other theories may have been considered. In the future authors may wish to explain why NPT was selected, whether in preference to or in conjunction with other theories, identifying limitations and strengths of NPT in relation to other theories or frameworks that assist in implementation development and evaluation. This would assist researchers in assessing the relative merits of NPT and alternative theoretical frameworks. Similarly, researchers may wish to expand on the challenges of adopting NPT. This would assist others in implementation planning, and in making decisions about whether normalisation of an intervention would be feasible in the real world, and potentially across contexts.

## Conclusion

This review finds that NPT is widely used and seemingly beneficial in the development and evaluation of implementation interventions across topics in UK primary care settings, particularly in the support and self-management of chronic and co-morbid health conditions. Recent research shows the potential of NPT as a prospective tool in the accumulation of whole-systems knowledge. NPT has been used less often to examine the perspective of patients and others receiving interventions, and this may warrant further exploration. Furthermore, reporting of authors’ justification for adopting NPT is recommended.

## Supplementary information

**Additional file 1.** Quality checklist results for the assessment of risk of bias.

## Data Availability

Not applicable.

## Abbreviations

CFS/ME: Chronic Fatigue Syndrome / Myalgic Encephalomyelitis
NHS: National Health Service
NPM: Normalisation Process Model
NPT: Normalisation Process Theory
RCT: Randomised Control Trial
UK: United Kingdom
