# Supplementary material for: Application of normalisation process theory in understanding implementation processes in primary care settings in the UK: a systematic review
Source: BMC Fam Pract. 2020 Mar 16;21:52. doi: 10.1186/s12875-020-01107-y (PMC7075013; doi:10.1186/s12875-020-01107-y)
Supplement: Supplementary file 1 — Additional file 1. Quality checklist results for the assessment of risk of bias. [file 12875_2020_1107_MOESM1_ESM.docx]

**Quality checklist results for the assessment of risk of bias using the Qualitative CASP tool**

| **Author (year)** | **Tool used** | **Statement of aims** | **Appropriate methodology** | **Research Design** | **Recruitment strategy** | **Data collection** | **P-R relationship** | **Ethical issues** | **Data analysis** | **Statement of findings** |
| --- | --- | --- | --- | --- | --- | --- | --- | --- | --- | --- |
| *Band et al.^23^ (2017)* | Qualitative CASP | Yes | Yes | Yes | N/A | Yes | N/A | N/A | Yes | Yes |
| *Bayliss et al.^24^  (2016)* | Qualitative CASP | Yes | Yes | Yes | Yes | Yes | Yes | Yes | Yes | Yes |
| *Blickem et al.^25^  (2014)* | Qualitative CASP | Yes | Yes | Yes | Yes | Yes | Can’t tell | Yes | Yes | Yes |
| *Bouamrane and Mair.^26^ (2013)* | Qualitative CASP | Yes | Yes | Yes | Yes | Yes | Can’t tell | Yes | Yes | Yes |
| *Bouamrane and Mair.^27^ (2014)* | Qualitative CASP | Yes | Yes | Yes | Yes | Yes | Can’t tell | Yes | Yes | Yes |
| *Browne et al^28^. (2014)* | Qualitative CASP | Yes | Yes | Yes | Yes | Yes | Can’t tell | Yes | Yes | Yes |
| *Carter et al^30^. (2016)* | Qualitative CASP | Yes | Yes | Yes | Yes | Yes | Yes | Yes | Yes | Yes |
| *Coupe et al.^31^  (2014)* | Qualitative CASP | Yes | Yes | Yes | Yes | Yes | Yes | Yes | Yes | Yes |
| *De Brún et al.^32^ (2015)* | Qualitative CASP | Yes | Yes | Yes | Yes | Yes | Can’t tell | Can’t tell | Yes | Yes |
| *Farr et al.^33^ (2018)* | Qualitative CASP | Yes | Yes | Yes | Yes | Yes | Can’t tell | Yes | Yes | Yes |
| *Grant et al.^34^ (2017)* | Qualitative CASP | Yes | Yes | Yes | Yes | Yes | Can’t tell | Yes | Yes | Yes |
| *Hoskins et al.^35^ (2016)* | Qualitative CASP | Yes | Yes | Yes | Yes | Yes | Can’t tell | Yes | Yes | Yes |
| *Kennedy et al.^36^ (2014)* | Qualitative CASP | Yes | Yes | Yes | Yes | Yes | Can’t tell | Yes | Yes | Yes |
| *Kennedy et al. ^37^ (2014)* | Qualitative CASP | Yes | Yes | Yes | Yes | Yes | Can’t tell | Yes | Yes | Yes |
| *Knowles et al. ^38^  (2013)* | Qualitative CASP | Yes | Yes | Yes | Yes | Yes | Can’t tell | Yes | Yes | Yes |
| **Author (year)** | **Tool used** | **Statement of aims** | **Appropriate methodology** | **Research Design** | **Recruitment strategy** | **Data collection** | **P-R relationship** | **Ethical issues** | **Data analysis** | **Statement of findings** |
| *Ling et al.^39^ (2012)* | Qualitative CASP | Yes | Yes | Yes | Yes | Yes | Can’t tell | Can’t tell | Yes | Yes |
| *Lionis et al.^40^ (2016)* | Qualitative CASP | Yes | Yes | Yes | Yes | Yes | Yes | Yes | Yes | Yes |
| *Martindale et al.^41^ (2017)* | Qualitative CASP | Yes | Yes | Yes | Yes | Yes | Yes | Yes | Yes | Yes |
| *Morden et al.^42^   (2015)* | Qualitative CASP | Yes | Yes | Yes | Yes | Yes | Yes | Can’t tell | Yes | Yes |
| *Morris et al.^43^   (2016)* | Qualitative CASP | Yes | Yes | Yes | Yes | Yes | Can’t tell | Can’t tell | Yes | Yes |
| *O’Donnell and Kaner.^44^ (2017)* | Qualitative CASP | Yes | Yes | Yes | Yes | Yes | Yes | Yes | Yes | Yes |
| *Ong et al.^45^  (2014)* | Qualitative CASP | Yes | Yes | Yes | Yes | Yes | Can’t tell | Yes | Yes | Yes |
| *Porter et al. ^46^  (2016)* | Qualitative CASP | Yes | Yes | Yes | Yes | Yes | Yes | Yes | Yes | Yes |
| *Reeve et al.^47^  (2016)* | Qualitative CASP | Yes | Yes | Yes | Yes | Yes | Yes | Yes | Yes | Yes |
| *Reeve et al.^48^ (2018)* | Qualitative CASP | Yes | Yes | Yes | Yes | Yes | Can’t tell | Yes | Yes | Yes |
| *Ricketts et al^49^.   (2016)* | Qualitative CASP | Yes | Yes | Yes | Yes | Yes | Can’t tell | Yes | Yes | Yes |
| *Rostami et al.^50^ (2018).* | Qualitative CASP | Yes | Yes | Yes | Yes | Yes | Can’t tell | Yes | Yes | Yes |
| *Stevenson^51^ (2015)* | Qualitative CASP | Yes | Yes | Yes | Yes | Yes | Can’t tell | Yes | Yes | Yes |
| *Teunissen et al.^52^ (2017)* | Qualitative CASP | Yes | Yes | Yes | Yes | Yes | Can’t tell | Yes | Yes | Yes |
| *Webster et al.^15^ (2016)* | Qualitative CASP | Yes | Yes | Yes | Yes | Yes | Can’t tell | Yes | Yes | Yes |

***Quality checklist results for assessment of risk of bias using the RCT CASP tool***

| **Author (year)** | **Addressed a clearly focussed issue** | **Randomised assignment** | **All trial participants properly accounted for** | **Participants blinded to treatment** | **Similar groups at outset of trial** | **Were control groups treated equally** | **Large treatment effect** | **Precise estimate of treatment effect** | **Results applicable to local context/population** | **All clinically important outcomes considered** |
| --- | --- | --- | --- | --- | --- | --- | --- | --- | --- | --- |
| *Buckingham et al.^29^  (2015)* | Yes | Yes | Yes | No | No | Yes | No | No | Yes | Yes |
